# The Candidate TB Vaccine, MVA85A, Induces Highly Durable Th1 Responses

**DOI:** 10.1371/journal.pone.0087340

**Published:** 2014-02-03

**Authors:** Michele Tameris, Hennie Geldenhuys, Angelique KanyKany Luabeya, Erica Smit, Jane E. Hughes, Samantha Vermaak, Willem A. Hanekom, Mark Hatherill, Hassan Mahomed, Helen McShane, Thomas J. Scriba

**Affiliations:** 1 South African Tuberculosis Vaccine Initiative, Institute of Infectious Disease and Molecular Medicine and School of Child and Adolescent Health, University of Cape Town, Cape Town, South Africa; 2 Jenner Institute, Nuffield Department of Clinical Medicine, University of Oxford, Oxford, United Kingdom; University of Delhi, India

## Abstract

**Background:**

Vaccination against tuberculosis (TB) should provide long-term protective immunity against *Mycobacterium tuberculosis* (*M.tb*). The current TB vaccine, Bacille Calmette-Guerin (BCG), protects against disseminated childhood TB, but protection against lung TB in adolescents and adults is variable and mostly poor. One potential reason for the limited durability of protection may be waning of immunity through gradual attrition of BCG-induced T cells. We determined if a MVA85A viral-vector boost could enhance the durability of mycobacteria-specific T cell responses above those induced by BCG alone.

**Methods:**

We describe a long-term follow-up study of persons previously vaccinated with MVA85A. We performed a medical history and clinical examination, a tuberculin skin test and measured vaccine-specific T cell responses in persons previously enrolled as adults, adolescents, children or infants into three different Phase II trials, between 2005 and 2011.

**Results:**

Of 252 potential participants, 183 (72.6%) consented and completed the study visit. Vaccine-induced Ag85A-specific CD4+ T cell responses were remarkably persistent in healthy, HIV-uninfected adults, adolescents, children and infants, up to 6 years after MVA85A vaccination. Specific CD4+ T cells expressed surface markers consistent with either CD45RA−CCR7+ central memory or CD45RA−CCR7− effector memory T cells. Similarly durable Ag85A-specific CD4+ T cell responses were detected in HIV-infected persons who were on successful antiretroviral therapy when MVA85A was administered. By contrast, Ag85A-specific CD4+ T cell frequencies in untreated MVA85A-vaccinated HIV-infected persons were mostly undetectable 3–5 years after vaccination.

**Conclusion:**

MVA85A induces remarkably durable T cell responses in immunocompetent persons. However, results from a recent phase IIb trial of MVA85A, conducted in infants from the same geographic area and study population, showed no vaccine efficacy, suggesting that these durable T cell responses do not enhance BCG-induced protection against TB in infants.

## Introduction

Despite all efforts, tuberculosis (TB) remains a major global problem, especially in Sub-Saharan Africa and Asia. Approximately 1.45 million people died from TB in 2011 [1]. It is clear that better intervention strategies against TB are urgently required. Epidemiological modelling suggests that global TB elimination targets can only be achieved with an effective vaccination strategy, coupled with better diagnosis and more effective treatment of persons with TB disease [2], [3].

Most researchers recognise that vaccination against TB should aim to induce a specific T cell response. However, it is not known which characteristics of a T cell response may mediate protection against TB [4], [5]. Vaccination with Bacille Calmette-Guerin (BCG) confers good protection against disseminated childhood TB, but provides variable protection against pulmonary disease, especially in adolescents and adults [6], [7]. One possible reason underlying limited protection beyond childhood is waning of BCG-induced immunity against TB through gradual attrition of BCG-induced T cell responses [8].

Successful prophylactic vaccines, such as those against tetanus toxoid [9], yellow fever [10] and smallpox [11] induce protective immunity that persists for decades. We proposed that vaccination against TB should aim to induce similarly long-lived immunity, in the form of mycobacteria-specific memory T cells. A heterologous prime–boost vaccination strategy may potentially improve the longevity of the BCG-induced T cell response. We sought to determine if a BCG-prime viral-vector vaccine boost strategy against TB, with Modified Vaccinia virus Ankara expressing the mycobacterial antigen Ag85A (MVA85A), could enhance the durability of mycobacteria-specific T cell responses above those induced by BCG alone. The MVA85A vaccine showed promising immunogenicity and induced protective immunity against *M.tb* in cattle [12], rhesus macaques [13], mice [14] and guinea pigs [15]. Phase I and IIa clinical trials of MVA85A have been conducted in healthy adults, adolescents and children from the United Kingdom, the Gambia, South Africa and Senegal [16]–[25]. Adult participants have included HIV infected people, both on antiretroviral (ARV) therapy and ARV naïve, and *M.tb*-infected participants. MVA85A was shown to be well tolerated in these different clinical populations from different settings. Further, the vaccine induced robust Ag85A-specific CD4 T cells responses, which predominantly expressed IFN-γ, TNF-α and IL-2, in vaccinees of all ages. However, a recent phase IIb proof-of-concept trial in South African infants showed no efficacy against TB [26].

Current approaches to clinical evaluation of novel TB vaccines, such as the ones listed above, typically limit follow-up of vaccinated participants to 6 or perhaps 12 months, precluding evaluation of long-lived immunity. We conducted a long-term follow-up (3–6 years) of persons who received MVA85A as a boost vaccine to routine BCG vaccination at birth. We re-enrolled previous participants of three phase I/IIa trials conducted between 2005 and 2011 in the Western Cape Province of South Africa, a setting where TB is endemic, and performed a medical history and clinical examination, a tuberculin skin test (TST) and measured the frequencies, phenotypes and cytokine expression profiles of antigen-specific T cell responses.

## Materials and Methods

### Study Design and Population

This was a descriptive cross-sectional study of the safety and immune responses induced by MVA85A in previous participants of phase I and II trials of MVA85A [17], [22]–[24]. These trials were conducted between 2005 and 2011 at the South African TB Vaccine Initiative Field Site in the Western Cape Province of South Africa. Previous participants included 24 healthy adults [17] and 12 healthy adolescents [22] enrolled into the TB008 trial (clinicaltrials.gov NCT00460590), 48 *M.tb* and/or HIV-infected adults enrolled into the TB011 trial (clinicaltrials.gov NCT00480558) [24] and 24 healthy children [22] and 144 healthy infants [23] enrolled into the TB014 trial (clinicaltrials.gov NCT00679159). All three trials were open label; in trial TB014 one in four infants was allocated to receive Prevenar® as a placebo control, instead of MVA85A. All participants except one in trial TB014 had completed per-protocol follow up, which was 12 months in TB008 and TB011 and 6 months in TB014. Previous participants or their parents/legal guardians were approached to participate in this follow up study. Written informed consent and assent, if the participant was a minor between 7 and 17 years old, were obtained in the participant's home language. Study procedures included a targeted medical history and examination, phlebotomy, and administration of a tuberculin skin test (TST, Mantoux method). Consent for participation included granting access to participant medical records including CD4+ T cell count and viral load records for trial TB011 participants. The TST was read after 48–72 hours at the participant's home, school or workplace using a transparent ruler to measure the largest transverse diameter [27]. For consistency with TST cut-offs with previous trials, we defined TST indurations of >15 mm as positive in adults and adolescents who were previous participants of trial TB008 [17], [22], whereas TST indurations of ≥10 mm were defined as positive in children, infants and *M.tb*- infected and/or HIV-infected adults who were participants of trials TB011 and TB014 [23], [24].

This study was approved by the University of Cape Town Health Sciences Faculty Human Research Ethics Committee (UCT FHS HREC) and the Oxford University Tropical Research Ethics Committee (OxTREC). All investigations were conducted according to the principles expressed in the Declaration of Helsinki.

### IFN-γ ELISpot assay

Antigens included a single pool of peptides spanning the Ag85A protein (15-mers, overlapping by 10 amino acids, each at 2 µg/mL; Peptide Protein Research Ltd) and live BCG (from the vaccine vial, strain SSI, Staten Serum Institute, 1.2×10^6^ CFU/mL), prepared as previously described [22]. A peptide pool spanning the *M.tb*-specific antigens ESAT-6 and CFP-10 (15-mers, overlapping by 10 amino acids; 2 µg/mL each, Peptide Protein Research Ltd) was also included. Medium alone served as negative control and phytohemagglutinin (PHA, Sigma-Aldrich, 10 µg/mL) as positive control. Plates, containing 3×10^5^ peripheral blood mononuclear cells (PBMC) per well, were incubated for 18 hours at 37°C and developed according to the manufacturer's protocol (Mabtech). Assays were performed in duplicate wells and the average (with background subtracted) was used for analysis. The cut-off for positive responses was 50 spot forming cells (SFC) per million PBMC [22].

### Whole blood intracellular cytokine staining

Briefly, 0.5 mL heparinized whole blood was incubated immediately after collection with antigens in the presence of anti-CD28 and anti-CD49d (0.5 µg/mL each, BD Biosciences). After 7 hours, Brefeldin A (Sigma-Aldrich) was added and samples were incubated for a further five hours. A single pool of Ag85A peptides (2 µg/mL per peptide) and BCG (1.2×10^6^ CFU/mL) were used as antigens. No antigen (co-stimulant antibodies only) was used as negative control and PHA (10 µg/mL,) as positive control. Erythrocytes were lysed and white cells fixed using FACSLysing Solution (BD Biosciences), before cryopreservation. Cells were thawed in batch, permeabilized with BD Perm/Wash buffer and stained with fluorescent antibodies. Antibodies for detecting cytokine responses by CD4+ and CD8+ T cells were as follows: CD3-Pacific Blue (UCTH1), CD8-PerCPCy5.5 (SK1), CCR7-PE (150503), IFN-γ-AlexaFluor700 (K3), IL-2-FITC (5344.111, all from BD Biosciences), TNF-α-PECy7 (MAb11), IL-17-AlexaFluor647 (eBio64CAP17, both from eBiosciences), CD45RA-BV570 (HI100, Biolegend) and CD4-QDot605 (SK3, Invitrogen). At least 1 million total cells were acquired on an LSR II flow cytometer (BD Biosciences). Cell doublets were excluded using forward scatter (FSC)–area versus FSC-height parameters. Single stained mouse κ beads were used to calculate compensations for every run.

### Statistical analyses

Descriptive statistics were used to analyse medical history and clinical examination. TST conversion was reported as either conversion from a negative to positive induration using original trial protocol–defined cut-offs (**Table 1**), or conversion from a negative to positive induration plus an increase in induration of ≥6 mm in trials TB008 and TB014. For each sub-group we recorded the occurrence of TB disease from the medical history and rates of *M.tb* infection, measured by IFN-γ ELISpot responses to ESAT-6 and CFP-10. A cut-off of 50 spot forming units (SFU) per million PBMC was considered positive, as previously described [17].

**Table 1:** Demographic and clinical characteristics recorded after long-term follow-up of previously MVA85A-vaccinated individuals.

	TB008 adults 5×10^7^ pfu (n = 17) [N = 24]	TB008 adolescents 5×10^7^ pfu (n = 9) [N = 12]	TB014 children 5×10^7^ pfu (n = 16) [N = 24]	TB014 infants	TB011						
				2.5×10^7^ pfu (n = 27) [N = 36]	5×10^7^ pfu (n = 29) [N = 36]	1×10^8^ pfu (n = 26) [N = 36]	Prevenar® (n = 21) [N = 36]	Group 1 M.tb-infected @5×10^7^ pfu (n = 11) [N = 12]	Group 2 HIV-infected 5×10^7^ pfu (n = 8) [N = 12]	Group 3 M.tb and HIV-infected 5×10^7^ pfu (n = 9) [N = 12]	Group 4 HIV-infected, on successful ARV 5×10^7^ pfu (n = 9) [N = 12]
Male gender, n (%)	5 (29.4%)	4 (44.4%)	10 (62.5%)	11 (40.7%)	15 (51.7%)	7 (26.9%)	12 (57.1%)	31.6 (31.3–53.5)	38.0 (27.4–46.9)	33.5 (20.6–45.1)	38.1 (30.8–46.9)
Age in years, mean (range)	41.5 (26.5–51.1)	18.9 (18.2–19.4)	8.0 (5.5–11.1)	4.1 (3.8–4.5)	3.9 (3.6–4.3)	3.4 (3.3–3.9)	3.8 (3.3–4.3)	32.35 (23.8–43.5)	30.6 (21.2–37.4)	26.6 (22.2–32.7)	30.6 (21.5–40)
Mean BMI, Kg/m^2^ (range)	31.8 (24.1–45.7)	28.2 (18.8–44.1)	16.6 (12.3–22.8)	15.5 (12.1–21)	15.0 (12.5–18.4)	14.2 (10.6–17.2)	15.5 (12.7–25.9)	4.2 (4.1–4.3)	3.7 (3.4–4.2)	3.1 (2.3–3.9)	2.0 (1.9–2.2)
Years since vaccination, mean (range)	5.7 (5.3–6.1)	4.6 (4.4–4.8)	3.7 (3.7–3.9)	3.3 (3.2–3.5)	3.15 (3.1–3.3)	2.9 (2.8–3.1)	3.1 (2.8–3.3)	N/A	0	0	1 (11.1%)
TST conversion, n*	1	2	1	2	2	4	0	N/A	N/A	N/A	N/A
TST conversion of ≥6 mm, n	0	2	1	2	2	4	0	N/A	N/A	N/A	N/A
Received TB treatment since vaccination, n	0	0	0	2	1	1	3	0	1	1	0
Received TB prophylaxis, n	N/A	N/A	0	1	1	2	2	0	0	0	0
ARV commenced during observation period, n (%)	N/A	N/A	N/A	N/A	N/A	N/A	N/A	0	3 (37.5%)	3 (33.3%)	N/A

@ Dose of MVA85A vaccine administered intradermally; pfu, plaque forming units.

* We applied TST cut-offs consistent with the original trial protocols, namely 15 mm in TB008, and 10 mm in TB014 and TB011.

N, participants in the original clinical trial; n, participants re-enrolled.

Data analysis was performed with FlowJo software version 9.0 (TreeStar). The Boolean gate platform was used with individual cytokine gates to create all possible response pattern combinations. The data analysis programs PESTLE (version 1.5.4) and SPICE (Simplified Presentation of Incredibly Complex Evaluations; version 4.1.6) were used to analyse flow cytometry data and generate graphical representations of T cell responses using background-deducted flow cytometric data (both kindly provided by Mario Roederer, Vaccine Research Center, NIAID, NIH).

Statistical tests were performed using Prism version 4.03 (GraphPad). T cell responses at different time points were compared using Kruskal-Wallis (overall effect) and Mann-Whitney U tests. Paired comparisons of immune responses at individual pre- or post-vaccination time points were done using the Wilcoxon Matched Pairs test.

## Results

### Participants

Of the 252 participants of the previous phase I/II MVA85A vaccine trials, 182 (72.2%) were located and enrolled into this long-term follow-up study.

Seventeen of 24 (70.8%) adults from trial TB008 were re-enrolled at a median of 5.7 years since MVA85A vaccination (range 5.3–6.1 years), while nine of 12 (75%) adolescents were re-enrolled at a median of 4.6 years (range 4.4–4.8 years) post-vaccination (**Table 1**). One hundred and nineteen (70.8%) of the 168 TB014 participants were located and enrolled, including 16 (66.6%) of 24 children and 103 of 144 (71.5%) infants. The infants were distributed across three groups who received increasing MVA85A doses, and a placebo control group, who received Prevenar®, as laid out in **Table 1**.

Of the 48 trial TB011 participants, 37 (77.1%) were located and enrolled (**Table 1**). These included 11 (91.6%) from Group 1 (*M.tb*-infected, HIV-negative), eight (66.7%) from Group 2 (*M.tb*-uninfected, HIV-infected), nine (75%) from Group 3 (*M.tb* and HIV co-infected) and nine (75%) from Group 4 (HIV-infected and stable on antiretroviral therapy (ARV); +/− M.tb infection). Follow-up time since MVA85A vaccination ranged from 1.9 to 4.3 years (**Table 1**).

### Health Assessment

Nine healthy babies were born to participants of the TB008 [17] and TB011 [24] trials since the final trial study visit. No hospitalisations or visits to a health facility for a condition possibly, probably or definitely related to the study vaccine were reported for any participants of the TB008 trial. None of the TB008 participants had been prescribed TB treatment since the trial. One adult and two adolescents may have acquired *M.tb*-infection as they recorded a TST reading exceeding 15 mm, the cut-off for *M.tb* infection in the TB008 trial. The increase in TST induration for these two adolescents was ≥6 mm.

Seven of the participants who previously enrolled as infants into the TB014 trial had received TB treatment since the final clinical trial visit: two in Group 1, one each in Groups 2 and 3, and three in the Prevenar® group. A total of 6participants who previously enrolled as infants received TB prophylaxis; one in each of Group 1 and 2, two in Group 3, and two Prevenar recipients received TB prophylaxis. TST conversion to >10 mm was reported in one of the older children, two participants each of infant Groups 1 and 2, four in Group 3 and none in the Prevenar® group (p = 0.06).

One participant in TB011 Group 4, who had a negative TST during the original trial, converted to a positive TST. A single participant in Group 2 and one in Group 3 had commenced TB treatment since the last study visit, while three Group 2 participants and three Group 3 participants had initiated antiretroviral therapy. All HIV-positive participants showed continued immunologic and virologic control as measured by CD4+ T cell counts and HIV RNA load. Two deaths had occurred in HIV-infected participants (Group 2), one due to uncontrolled epilepsy and one due to unknown natural causes. These were not considered related to the study vaccine.

### MVA85A-induced Ag85A-specific T cell responses are remarkably durable

**Figure 1:**
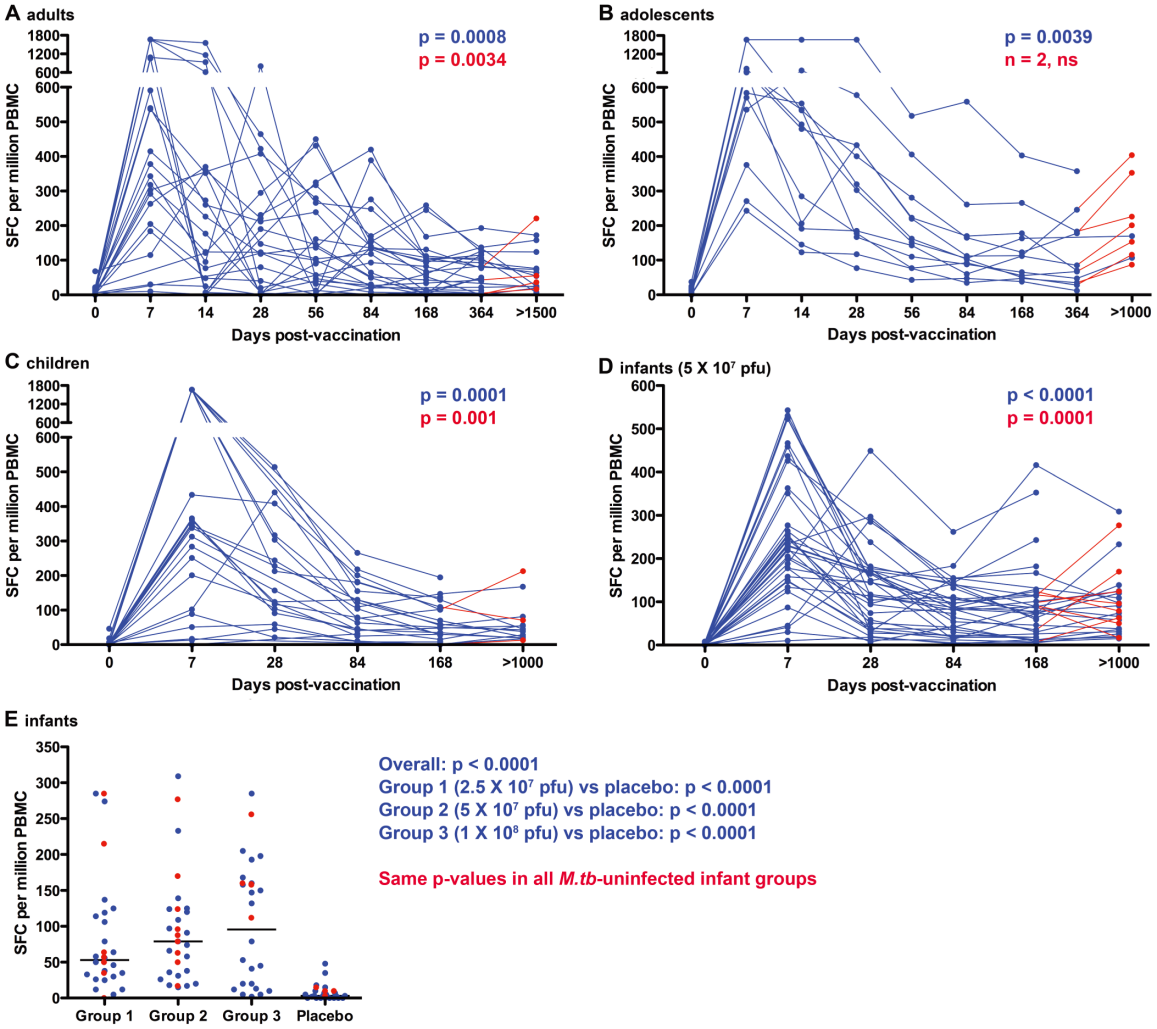
Longitudinal tracking of Ag85A-specific IFN-γ ELISPOT responses in subjects vaccinated with MVA85A 3 to 6 years ago. Red data points denote measurements from individuals who became infected with *M.tb* since completion of the original clinical trial. *M.tb* infection was defined as conversion to a positive IFN-γ ELISpot assay response to ESAT-6/CFP-10. Frequencies of IFN-γ spot forming cells (SFC) detected before vaccination and at the last time point after MVA85A vaccination were compared using the Wilcoxon signed rank test. Blue p-values denote comparison of data from all individuals, while red p-values denote comparison of *M.tb*-uninfected (negative responders to ESAT-6/CFP-10) individuals only. (**A–D**) Longitudinal tracking of Ag85A-specific T cell responses in adults (**A**, n = 17), adolescents (**B**, n = 9), children (**C**, n = 15) and infants (**D**, n = 27), who received 5×10^7^ plaque forming units (pfu) of MVA85A. (**E**) Comparison of Ag85A-specific IFN-γ ELISPOT responses in subjects who were vaccinated more than 3 years ago with different doses of MVA85A as infants, or who received the placebo vaccine, Prevenar (n = 24 for Group 1, n = 27 for Group 2; n = 24 for Group 3 and n = 23 for the placebo Group). The overall effect was calculated using the Kruskal-Wallis test, while responses in each dose group were compared to the placebo group using the Mann-Whitney U test. Median and IQR IFN-γ ELISPOT response values are shown in **Table S1**.

To determine if MVA85A-induced immune responses persisted for years, we measured the magnitude of Ag85A-specific T cell responses by IFN-γ ELISpot assay in peripheral blood collected 3–6 years after MVA85A vaccination. The MVA85A vaccine trials in adults, adolescents and children did not include placebo groups, precluding comparison of response durability in vaccinated and unvaccinated groups. We therefore compared response magnitudes detected after long-term follow-up with those detected before MVA85A vaccination. Frequencies of Ag85A-specific IFN-γ-expressing T cells detected 3 to 6 years after MVA85A vaccination of adults, adolescents, children and infants were still significantly higher than those detected before vaccination (**Figure 1A–D** and **Table S1**). Because the study design for the TB014 infant trial included a placebo group (Prevenar®), we also compared magnitudes of Ag85A-specific T cells detected in each vaccine dose group to the placebo group. Frequencies of IFN-γ-expressing Ag85A-specific T cells detected ±3 years after vaccination with either 2.5×10^7^, 5×10^7^ or 10×10^7^ plaque-forming units (pfu) of MVA85A significantly exceeded those detected in placebo recipients, most of whom had undetectable Ag85A-specific T cell responses (**Figure 1E**).

Given the high levels of exposure to *M.tb* in the TB endemic setting where these trials were conducted, we reasoned that Ag85A-specific immune responses may have been augmented in study participants who had acquired *M.tb* infection since the original vaccine trials. We therefore excluded participants with positive IFN-γ ELISpot responses to ESAT-6/CFP-10 from these analyses (persons found to be *M.tb*-infected at screening were excluded from the original TB008 and TB014 trials). Frequencies of Ag85A-specific T cells after long-term follow-up of adults, children and infants without *M.tb* infection were still significantly higher than those detected before vaccination (red p-values in **Figure 1A, C–E**). Only two participants in the adolescent group remained uninfected; comparison of responses in uninfected persons within this group was therefore not possible (**Figure 1B**).

### Persistent Ag85A-specific CD4 T cells are mostly polyfunctional

**Figure 2:**
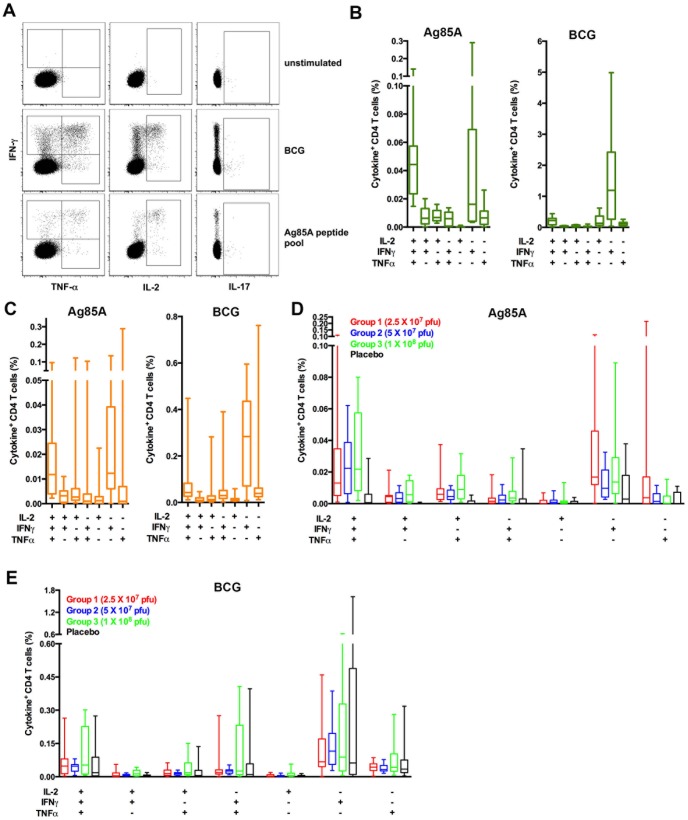
Characterization of Ag85A-specific CD4 T cell Th1 cytokine expression patterns after long-term follow-up, 3–5 years after MVA85A vaccination. (**A**) Representative gating of CD4 T cell expression of IFN-γ, IL-2, TNF-α and/or IL-17 in blood left unstimulated, or stimulated with BCG or Ag85A peptide pool, collected 1,187 days post-vaccination from a single participant who was 5 months old when originally vaccinated. (**B–E**) Frequencies of cytokine-expressing Ag85A or BCG-speciific CD4 T cells were measured by whole blood intracellular cytokine staining assay in adolescents (**B**), children (**C**) and infants (**D** and **E**). Cytokine expression patterns in adolescents and children who acquired *M.tb*-infection and those who remained uninfected were not different (data not shown); all individuals, irrespective of *M.tb*-infection status are shown in **B** (n = 9) and **C** (n = 16). Cytokine expression patterns in all infants (**D** and **E**, n = 15, 11, 12 and 11 for groups 1, 2, 3 and placebo respectively) were also not significantly different. For all box and whisker plots, horizontal lines represent medians, boxes represent the IQR and whiskers represent the range for each group of participants.

We previously showed that Ag85A-specific CD4+ T cells detected 6–12 months after MVA85A vaccination in adolescents, children and infants were predominantly polyfunctional, co-expressing the Th1 cytokines IFN-γ, TNF-α, and IL-2 [22], [23]. To determine if this cytokine expression profile changed over time, we characterised the cytokine expression profile of persistent Ag85A-specific CD4+ T cells by flow cytometry (**Figure S1 and** **Figure 2**). Ag85A-specific T cells detected 3–6 years after MVA85A vaccination of adolescents predominantly resided in the IFN-γ, TNF-α and IL-2 co-expressing, polyfunctional CD4+ T cell subset (**Figure 2B**). A considerable proportion of Ag85A-specific T cells also expressed IFN-γ alone. By comparison, BCG-specific CD4 T cells predominantly expressed IFN-γ alone. The cytokine expression profiles of persistent Ag85A-specific CD4+ T cells detected in previously vaccinated children and infants were very similar, while BCG-specific CD4 T cells in these younger age groups also predominantly expressed IFN-γ alone (**Figure 2C and D**). We did not observe a marked effect of underlying *M.tb* infection on this cytokine expression profile; the same polyfunctional and IFN-γ+ cells dominated the Ag85A-specific response when *M.tb*-infected donors were excluded (**Figure S2A**).

Longitudinal analysis of the dominant IFN-γ, TNF-α and IL-2 expressing, polyfunctional CD4+ T cell subset in adolescents, children and infants confirmed the findings made by ELISpot assay: responses detected after long-term follow-up were still significantly higher than those detected before vaccination (**Figure S2 B–D**).

We also sought to characterise cytokine expression profiles of antigen-specific CD8+ T cells by flow cytometry. However, as previously reported [17], [22]–[24], Ag85A-specific CD8+ T cells were mostly undetectable, or frequencies were too low to characterise, in participants from all study groups (data not shown).

### Persistent Ag85A-specific CD4+ T cells predominantly express an effector memory phenotype

**Figure 3:**
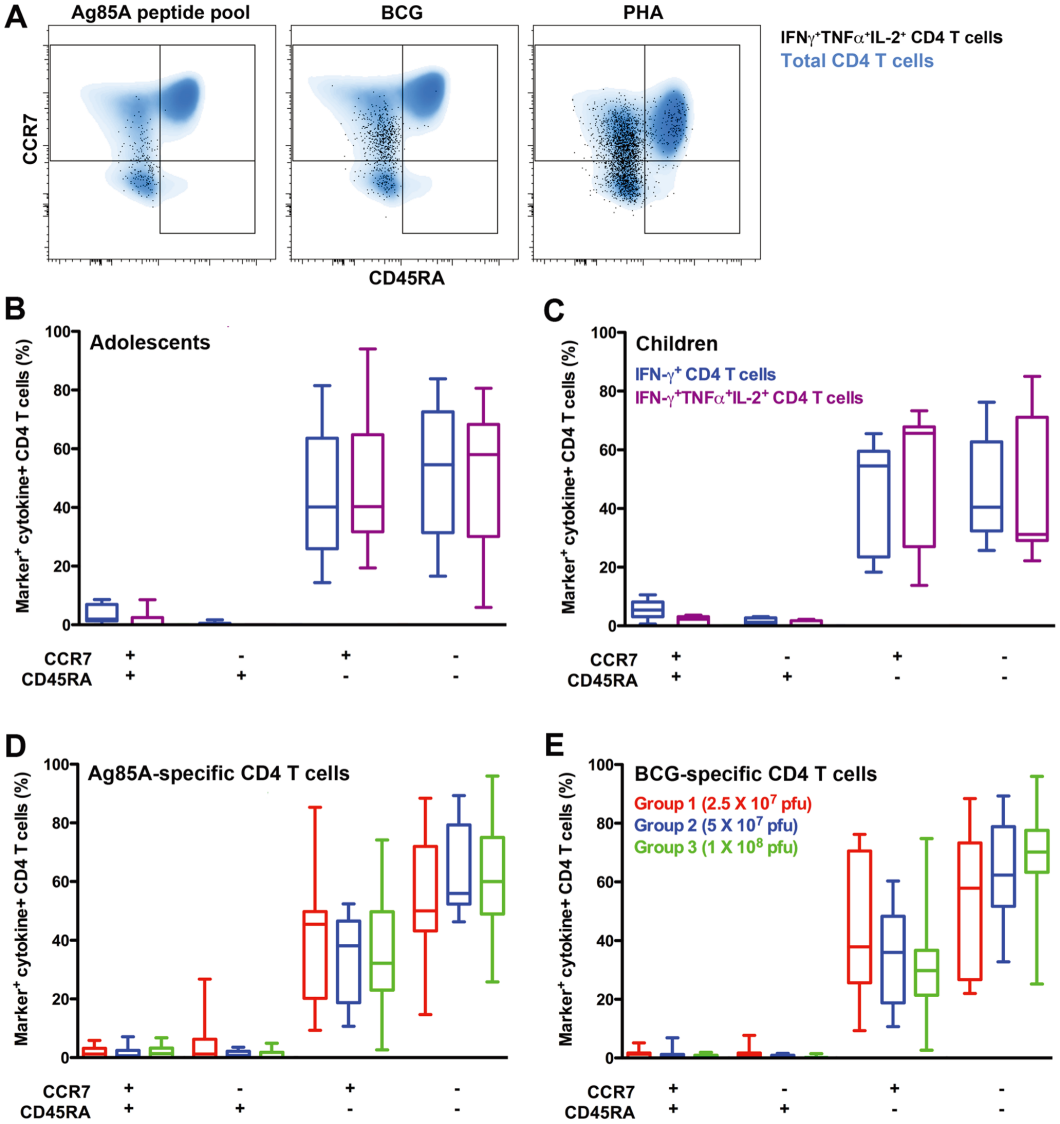
Memory phenotype of highly persistent Ag85A-specific CD4 T cells. (**A**) Representative flow cytometry overlay plots, from a MVA85A-vaccinated child, showing CD45RA and CCR7 co-expression patterns by the entire CD4 T cell subset (blue) and Ag85A-specific CD4 T cells co-expressing IFN-γ, TNF-α and IL-2 (black dots) after in vitro stimulation with Ag85A peptides, live BCG or PHA. (**B**–**D**) Proportions of cytokine-expressing Ag85A-specific CD4 T cells expressing the indicated combination of CD45RA and CCR7, measured 3–5 years after MVA85A vaccination in adolescents (**B**, n = 9), children (**C**, n = 16) and infants (**D**, n = 15, 11 and 12 for groups 1, 2 and 3, respectively). (**E**) Proportions of cytokine-expressing BCG-specific CD4 T cells expressing the indicated combination of CD45RA and CCR7 in infants (n = 15, 11 and 12 for groups 1, 2 and 3, respectively). For all box and whisker plots, horizontal lines represent medians, boxes represent the IQR and whiskers represent the range for each group of participants.

Given the persistence of MVA85A-induced CD4+ T cells, we sought to determine the memory phenotype of the Ag85A-specific memory response. Long-lived central memory CD4+ cells typically express high levels of CCR7, which endows these CD45RA− memory cells with lymph node homing capacity [28]. By contrast, effector memory cells have lost expression of CCR7, reside in the periphery and migrate to sites of infection. Th1 cytokine-expressing Ag85A-specific and BCG-specific memory CD4+ T cells from all age groups were virtually exclusively CD45RA-negative (**Figure 3**). Ag85A-specific CD4+ cells displayed either a CCR7+CD45RA− central memory or CCR7−CD45RA− effector memory phenotype in adolescents and children (**Figure 3B and C**), while in infants the specific response predominantly displayed a CCR7−CD45RA− effector memory phenotype (**Figure 3D**). The latter phenotype was very similar to BCG-specific Th1 cytokine-expressing CD4+ T cells from infants, which also predominantly displayed an effector memory phenotype (**Figure 3E**). Underlying *M.tb* infection had no marked effect on the memory phenotypes of these cytokine-expressing Ag85A-specific CD4+ T cells in any of the groups (data not shown).

### Highly durable Ag85A-specific T cell responses in ARV-treated, but not untreated, HIV-positive vaccinees

**Figure 4:**
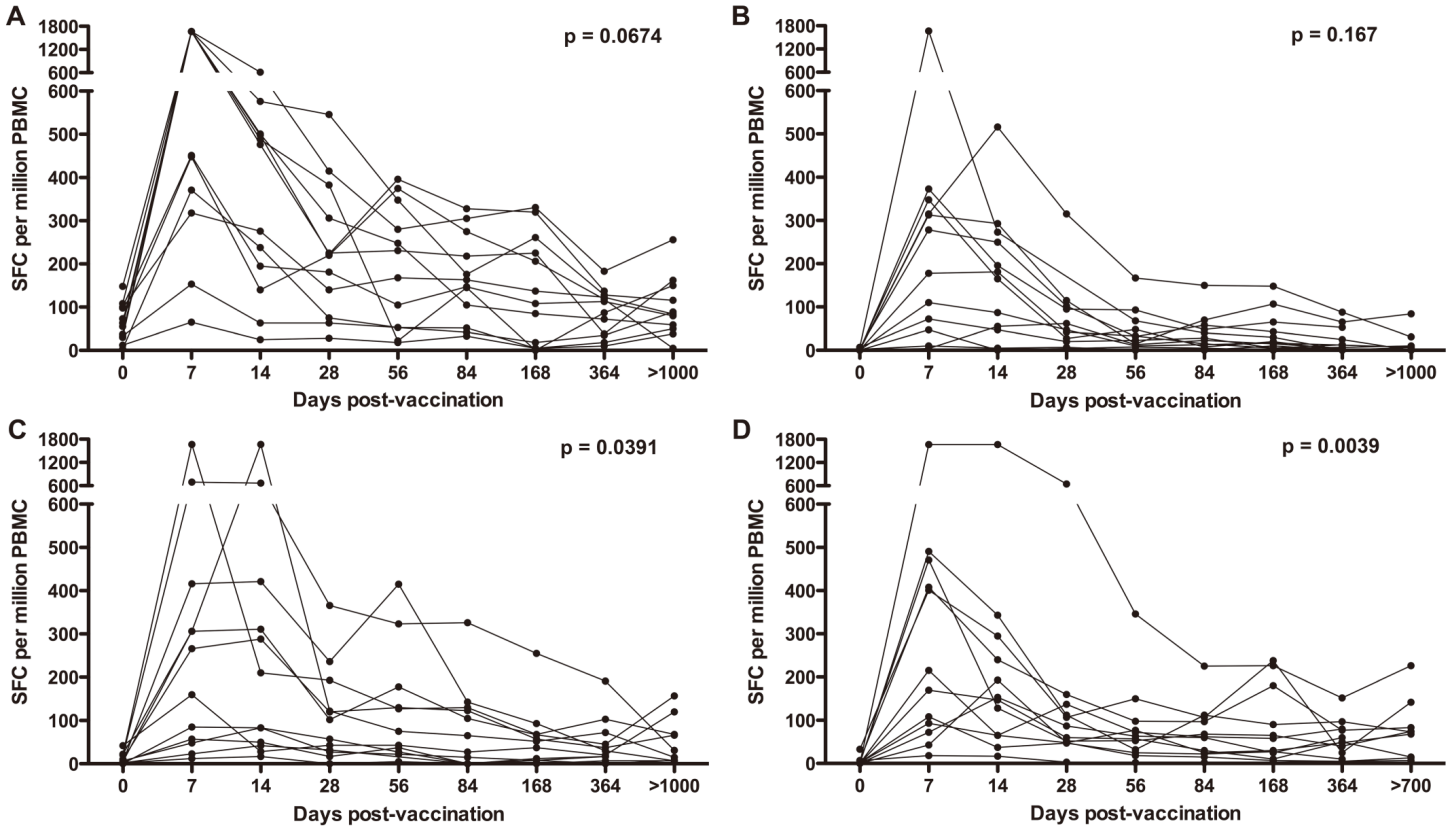
Longitudinal tracking of Ag85A-specific IFN-γ ELISPOT responses in *M.tb*-infected and/or HIV-infected subjects, who were vaccinated with MVA85A 2 to 6 years ago as part of the TB011 trial [24]. (**A**) *M.tb*-infected, HIV-negative individuals (n = 11); (**B**) *M.tb*-uninfected, HIV-infected individuals (n = 8); (**C**) *M.tb* and HIV-co-infected individuals (n = 9); (**D**) HIV-infected individuals on successful ART (irrespective of *M.tb*-infection status, n = 9). Frequencies of IFN-γ spot forming cells (SFC) detected before vaccination and at the last time point after MVA85A vaccination were compared using the Wilcoxon signed rank test. Median and IQR IFN-γ ELISPOT response values are shown in **Table S1**.

We also determined if MVA85A-induced immune responses persisted in participants of the TB011 trial, which was conducted in *M.tb*-infected, HIV-infected, co-infected persons and HIV-infected persons on successful ART [24]. Response magnitudes detected by IFN-γ ELISpot assay after long-term follow-up of these 4 study groups were compared with those detected before MVA85A vaccination. Frequencies of Ag85A-specific IFN-γ-expressing T cells in MVA85A vaccinated *M.tb*-infected (Group 1) or HIV-infected (Group 2) adults did not persist at magnitudes higher than those detected before vaccination (**Figure 4A and B** and **Table S1**). By contrast, Ag85A-specific response magnitudes persisted at levels exceeding pre-vaccination levels in the *M.tb*/HIV co-infected group (Group 3) and, in the ARV-treated, HIV-positive group (Group 4; **Figure 4C and D** and **Table S1**). Notably, Ag85A-specific T cells at long-term follow-up in untreated HIV-infected participants were largely undetectable (**Figure 4B**). These persons were not on ARV at the time of vaccination because their CD4 counts exceeded 350 cells/mm^3^. In contrast to the long-term memory responses, similar frequencies of Ag85A-specific responses were detected during the first month after MVA85A vaccination in the untreated and ARV-treated HIV-infected groups (**Figure 4B and D**).

Next, we characterised the cytokine expression profile of persistent Ag85A-specific CD4 T cells in participants of the TB011 trial. No marked differences in cytokine expression patterns of Ag85A-specific T cells detected 2–5 years after MVA85A vaccination were observed between the 4 groups (**Figure S3**). As observed for the other participant groups (**Figure 2**), Ag85A-specific T cells predominantly resided in the IFN-γ, TNF-α and IL-2 co-expressing, polyfunctional CD4+ T cell subset, while cells expressing IFN-γ only were also observed (**Figure S3**).

### High frequencies of Ag85A-specific T cells during the early response predict long-term persistence

**Figure 5:**
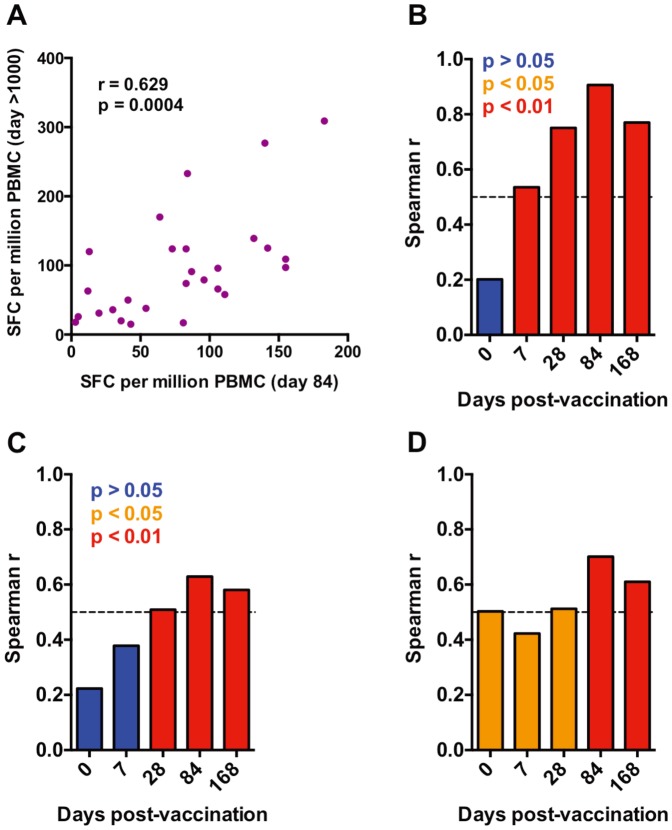
Early MVA85A-induced Ag85A-specific T cell responses predict the level of persisting Ag85A-specific T cell responses. (**A**) Representative Spearman correlation analysis of the frequencies of Ag85A-specific T cells detected by IFN-γ ELISpot assay on day 84 post-vaccination and more than 3 years post-vaccination in infants who received 5×10^7^ pfu of MVA85A. (**B**–**D**) Plots showing the Spearman r values obtained from correlation analyses between the frequencies of Ag85A-specific T cell responses detected after long-term follow-up (more than 1000 days post-vaccination), and those detected before MVA85A vaccination (day 0), or at 7, 28, 84 and 168 days MVA85A vaccination. Blue bars denote Spearman r values with a p-value above 0.05, orange bars denote p-values between 0.05 and 0.01 and red bars denote p-values below 0.01. (**B**) Infants who received 2.5×10^7^ pfu of MVA85A; (**C**) Infants who received 5×10^7^ pfu of MVA85A; (**D**) Infants who received 1×10^8^ pfu of MVA85A.

Finally, we sought to determine if frequencies of specific T cells during the first few months after MVA85A vaccination were associated with specific T cell frequencies that persisted for years after vaccination. We reasoned that such associations would indicate whether early T cell response magnitudes predict long-term persistence, suggesting that expensive and time-consuming long-term follow-up of vaccinees may not be necessary. We restricted these analyses to the TB014 infant groups, since the limited sample sizes of the other age groups did not allow definitive analyses. Frequencies of Ag85A-specific IFN-γ-expressing T cells detected 84 days after vaccination with 5×10^7^ pfu of MVA85A correlated directly with those detected >1000 days after vaccination (**Figure 5A**). Significant, direct correlations between persistent Ag85A-specific T cells responses and those measured 28, 84 and 168 days after MVA85A vaccination were observed in all 3 infant groups (**Figure 5B–D**). Responses measured 84 and 168 days post-vaccination most strongly correlated with frequencies of Ag85A-specific T cells detected >1000 days after vaccination, while pre-vaccination responses to Ag85A correlated poorly or not at all.

## Discussion

The aim of vaccination is to induce long-lasting adaptive immune responses that mediate rapid and effective anti-microbial immunity in the event of future pathogen exposure. To assess long-term health of vaccinees and longevity of memory T cell responses induced by a heterologous BCG-prime, MVA85A-boost vaccination strategy, we performed a follow-up of individuals who received a single MVA85A vaccination 2–6 years ago.

No major medical events or health-related issues that may relate to MVA85A were reported or discovered in any of the vaccinees. This was not unexpected, given previous experience that MVA85A is generally well tolerated; no vaccine-related serious adverse events have to date been reported in any of the open-label trials [17], [22]–[24]. Sample sizes were too small to definitively evaluate the significance of the reported cases of TB in this population, which resides in a setting of very high exposure to TB. Both cases of TB recorded in adults were in HIV-positive individuals, a group with an extremely high annual risk of TB disease [29]. We also observed several conversions to positive TST, and/or positive ELIspot responses to ESAT-6/CFP-10, during the follow-up period. New *M.tb* infection is not unexpected in this high burden setting and participant numbers were too small and study designs not appropriate to assess how MVA85A vaccination may have an impact on *M.tb* infection. The TST conversion rate of 8/103 (7.8%) observed in participants previously vaccinated as infants was lower than the recorded Quantiferon conversion rate of 163/2253 (7.2%) in a recent Phase IIb efficacy trial of MVA85A in infants at this site [26]. Regardless, no vaccine efficacy against *M.tb* infection was observed in that study.

We report remarkable persistence of MVA85A-induced Ag85A-specific CD4+ T cell responses in healthy, HIV-uninfected adults, adolescents, children and infants. Induction of such long-lived T cell responses is consistent with the finding that poxvirus-specific CD4+ T cells were detected decades after DryVax vaccination against smallpox [30]. Our data are congruent with and extend those reported by Odutola *et al.*, who observed durable T cell responses to MVA85A vaccination up to fourteen months post-vaccination in Gambian infants [20]. Long-term durability of immune responses has only been described for one other novel TB vaccine in preclinical studies [31] and one in clinical trials [32]. It is important to perform extended follow-up to measure longevity of vaccine-induced immunity, since vaccines should ideally provide protection against pathogen exposure for many years. A gradual attrition of mycobacteria-specific T cells has been hypothesised to underlie the waning of BCG-induced protection against pulmonary TB [8]. In light of this hypothesis, the durability of MVA85A-induced Ag85A-specific memory responses could be interpreted as a highly favourable outcome. However, we recently reported that MVA85A vaccination afforded no enhancement in protective efficacy against TB disease or *M.tb* infection in infants previously vaccinated with BCG, when given as a heterologous boost vaccine [26]. The reasons underlying the lack of efficacy over and above newborn BCG vaccination in this infant trial are not known and are currently under scrutiny. Regardless, given our present results on response durability, Ag85A-specific CD4+ T cell responses would have persisted well beyond the clinical follow-up period in the phase IIb efficacy trial. This suggests that attrition of Ag85A-specific memory T cell responses is unlikely to underlie the lack of vaccine protection against TB. It is possible that the different cytokine expression profile of Ag85A-specific (mostly IFN-γ^+^TNF-α^+^IL-2^+^) and BCG-specific (mostly IFN-γ^+^ alone) CD4+ T cell responses may confer differential immunity. Many investigators have hypothesized that polyfunctionality may be a favourable property of the T cell response (ref 5). However, the predominantly monofunctional IFN-γ^+^ CD4+ T cell response induced by BCG, which is known to confer at least partial efficacy against TB, provides data against this hypothesis. Many other factors including the choice of antigen, magnitude, tissue location, function and/or phenotype of the induced T cell response, and/or epidemiological or clinical factors which may include route and/or age of administration, dose of the vaccine and the high rate of *M.tb* transmission in the trial population may underlie the lack of vaccine-induced protection [26], [33].

Our results from untreated HIV-infected persons showed that Ag85A-specific CD4+ T cells were mostly undetectable 3–5 years after MVA85A vaccination. These persons were not on ARV at the time of vaccination because their CD4+ T cell counts exceeded 350 cells/mm^3^ at that time. By contrast, HIV-infected persons who were on successful ARV when they received MVA85A had readily detectable Ag85A-specific CD4+ T cells at their long-term follow-up visit, at levels exceeding those detected pre-vaccination. These results suggest that ARV may preserve or restore mechanisms required for induction of long-term T cell memory, which appear to be dysfunctional in untreated HIV infection, even at CD4+ T cell counts exceeding 350 cells/mm^3^. Our data support the recent recommendation to increase the CD4+ T cell count at which ARV should be initiated from 350 to 500 cells/mm^3^ [34].

Interestingly, similar frequencies of early effector Ag85A-specific responses were detected during the first month after MVA85A vaccination in persons with untreated and ARV-treated HIV-infection. This suggests that HIV-associated immune dysfunction may not affect acute effector responses as markedly as its detrimental impact on memory function.

Ag85A-specific CD4+ T cells detected after long-term follow-up expressed surface markers consistent with either CD45RA−CCR7+ central memory and CD45RA−CCR7− effector memory phenotypes. We expected persistence of vaccine-induced specific CD45RA−CCR7+ central memory T cells, given the well-described prominence of this long-lived memory subset in scenarios of cleared infections or vaccination [28], but observed an age-specific difference. In older individuals Ag85A-specific CD4+ T cells displayed a mixed central and effector memory phenotype whereas in infants these cells predominantly displayed an effector memory phenotype, which was similar to BCG-specific CD4+ T cells. Sort lived effector memory cells with immediate effector function are typically observed during the peak of the immune response, or in the context of persistent antigen load [28]. Our finding that the memory phenotype of Ag85A-specific CD4+ T cells was not significantly different in *M.tb*-infected and uninfected persons suggests that Ag85A-specific CD4+ T cell phenotypes are not overtly sensitive to persistent *M.tb* antigen load. Interestingly, induction and maintenance of effector memory T cells, by chronic antigen stimulation, has been proposed as a vaccination strategy against chronic infections [35], including *M.tb*. Since effector cells preferentially home to peripheral sites of inflammation such as the lung, and possess immediate effector functions, these cells are hypothesised to confer better protection against *M.tb* than central memory T cells. The partially protective effect of murine BCG vaccination against *M.tb* is consistent with this: BCG establishes a persistent infection in mice [36] and thus maintains a population of effector memory T cells [37].

Our study had a number of limitations. We used different cut-off values of TST induration to define a positive TST in participants who were previously enrolled into the TB008 trial and those who were previously enrolled into trials TB011 and TB014. The reason for this was based on our decision to apply the methods used in the previously completed trials, in which different TST induration cut-off values were applied. Regardless, identification of *M.tb*-infected participants by TST correlated well with identification of these participants by IFN-γ-expressing T cell responses to ESAT-6/CFP-10, measured by ELISpot assay. We are therefore confident that these technical factors have not affected our results to a significant degree.

Differences in intercurrent acquisition rates of *M.tb*-infection in different age groups limited our analyses of age-specific immune responses. For example, most adolescents (7 out of 9) had acquired *M.tb*-infection, while the majority of infants did not. Another limitation was the absence of placebo control groups in the TB008 and TB011 trials, which precluded comparison of antigen-specific T cell response durability in vaccinated and unvaccinated groups. However, data from the infant trial suggests placebo responses to Ag85A peptides would have been at the baseline level. Our experience highlights the value of a placebo group even in small phase I and II trials.

In summary, MVA85A induces remarkably durable Ag85A-specific T cell responses in immunocompetent persons. However, no vaccine efficacy was observed in a Phase IIb efficacy trial of MVA85A conducted in infants from the same geographic area and study population [26]. Taken together, our work suggests that durability of BCG-induced Ag85A-specific T cell response, which was significantly augmented by MVA85A vaccination, is not sufficient to enhance BCG-induced protection against TB in infants.

## Supporting Information

Figure S1 **Flow cytometric analysis of MVA85A-induced T cell cytokine production, measured by whole blood intracellular cytokine staining.** (**A**) Representative dotplots illustrating the gating strategy used to identify CD4 and CD8 T cells 1,187 days post-vaccination from a single participant who was 5 months old when originally vaccinated. Top plots, from left to right, white cells from whole blood were acquired and small, CD3+ T lymphocytes selected. Cell doublets were then excluded using forward scatter-area and forward scatter-height parameters, followed by selection of cells with consistent fluorescent signal during acquisition time (time gate). (**B**) Selection of CD4 T cells by first gating on CD8-negative cells, and then CD4+ T cells. This strategy, in which cytokine-expressing cells are plotted against the T cell marker, was used to ensure that downregulation of the CD4 or CD8 proteins by activated, cytokine+ T cells does not lead to exclusion of cytokine-expressing cells. (**C**) Selection of CD8 T cells by first gating on CD4-negative cells, and then CD8+ T cells.
(EPS)

Figure S2 (**A**) Frequencies of Ag85A-specific CD4 T cells detected by intracellular cytokine staining in infants who remained *M.tb* uninfected (n = 15, 11, 12 and 11 for groups 1, 2, 3 and placebo respectively). (**B–D**) Longitudinal frequencies of Ag85A-specific polyfunctional IFN-γ^+^TNF-α^+^IL-2^+^ CD4 T cells in adolescents (**B**), children (**C**) and (**D**) infants. P-values represent a comparison between the pre-vaccination and long-term follow-up responses, using the Mann-Whitney U test. For all box and whisker plots, horizontal lines represent medians, boxes represent the IQR and whiskers represent the range for each group of participants.
(TIF)

Figure S3 **Characterization of Ag85A-specific CD4 T cell Th1 cytokine expression patterns after long-term follow-up, 2–5 years after MVA85A vaccination in participants of the TB011 trial.** Frequencies of cytokine-expressing CD4 T cells were measured by whole blood intracellular cytokine staining assay. Red, *M.tb*-infected, HIV-negative individuals (n = 11); Black, *M.tb*-uninfected, HIV-infected individuals (n = 8); Blue, *M.tb* and HIV-co-infected individuals (n = 9); Green, HIV-infected individuals on successful ART (irrespective of *M.tb*-infection status, n = 8). For all box and whisker plots, horizontal lines represent medians, boxes represent the IQR and whiskers represent the range for each group of participants.
(EPS)

Table S1 **Medians and interquartile ranges of Ag85A-specific IFN-γ Elispot responses.**
(DOC)
