# Supplementary figures and images for: The Candidate TB Vaccine, MVA85A, Induces Highly Durable Th1 Responses
Source: PLoS One. 2014 Feb 3;9(2):e87340. doi: 10.1371/journal.pone.0087340 (PMC3911992; doi:10.1371/journal.pone.0087340)

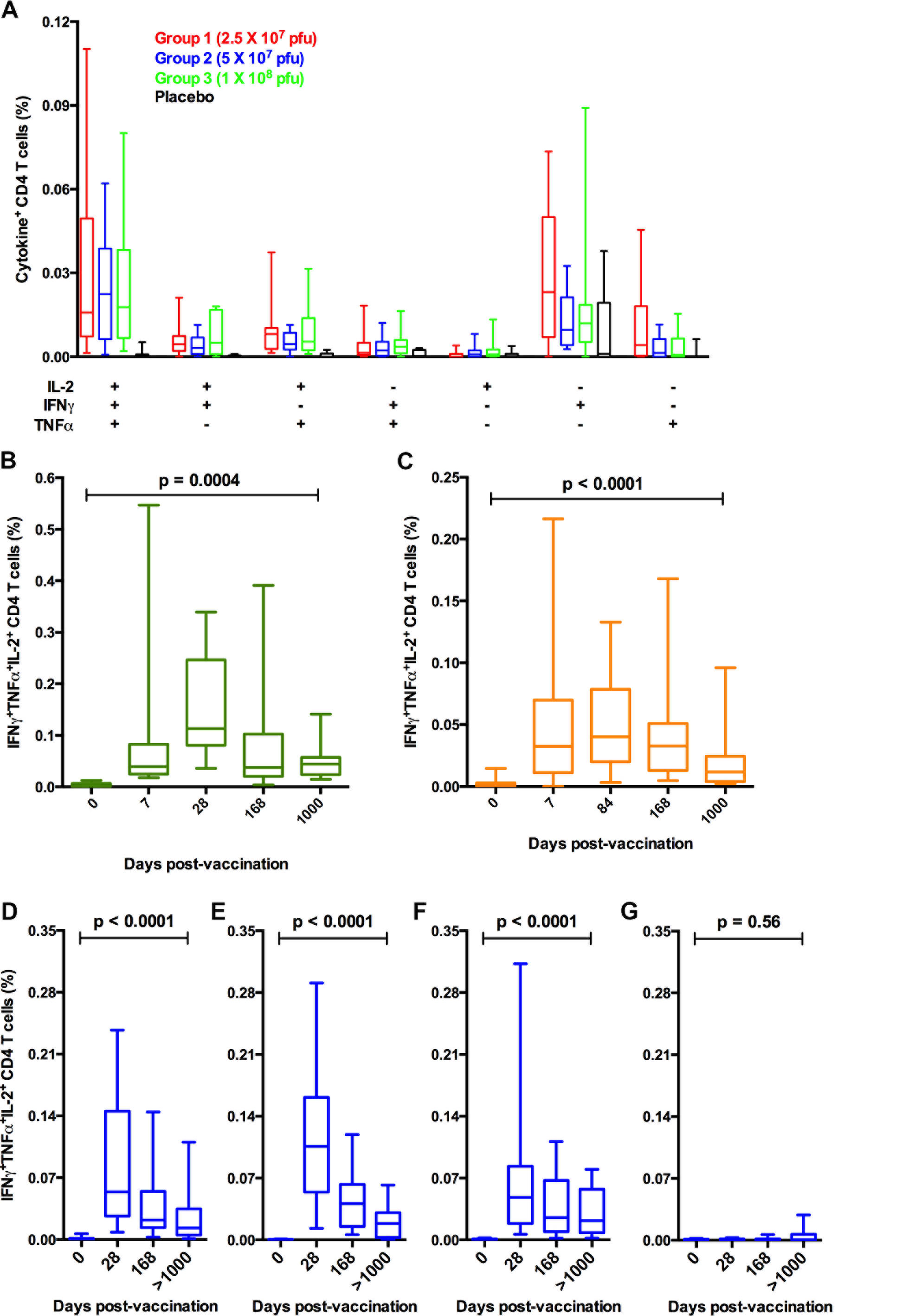

Supplement: Figure S2 — (A) Frequencies of Ag85A-specific CD4 T cells detected by intracellular cytokine staining in infants who remained M.tb uninfected (n = 15, 11, 12 and 11 for groups 1, 2, 3 and placebo respectively). (B–D) Longitudinal frequencies of Ag85A-specific polyfunctional IFN-γ+TNF-α+IL-2+ CD4 T cells in adolescents (B), children (C) and (D) infants. P-values represent a comparison between the pre-vaccination and long-term follow-up responses, using the Mann-Whitney U test. For all box and whisker plots, horizontal lines represent medians, boxes represent the IQR and whiskers represent the range for each group of participants. (TIF) [file pone.0087340.s002.tif]
