# Supplementary material for: The Candidate TB Vaccine, MVA85A, Induces Highly Durable Th1 Responses
Source: PLoS One. 2014 Feb 3;9(2):e87340. doi: 10.1371/journal.pone.0087340 (PMC3911992; doi:10.1371/journal.pone.0087340)
Supplement: Table S1 — Medians and interquartile ranges of Ag85A-specific IFN-γ Elispot responses. (DOC) [file pone.0087340.s004.doc]

**Supplementary Table 1:** Medians and interquartile ranges of Ag85A-specific IFN-γ Elispot responses.

|  | TB008 adults | | TB008 adolescents | | TB014 children | | TB014 infants | | TB011 | |  | | | | | | |
| --- | --- | --- | --- | --- | --- | --- | --- | --- | --- | --- | --- | --- | --- | --- | --- | --- | --- |
|  | |  | |  | |  | | Group 1 | | Group 2 | | Group 3 | Placebo | Group 1 | Group 2 | Group 3 | Group 4 |
| Day 0 | | 12 (2-20) | | 12 (1-19) | | 2 (0-5) | | 0 (0-5) | | 0 (0-3) | | 2 (0-5) | 0 (0-3) | 59 (31-100) | 0 (0-4) | 6 (3-22) | 1 (0-6) |
| Day 7 | | 361 (200-1060) | | 596 (416-707) | | 348 (127-1667*) | | 254 (108-352) | | 230 (151-360) | | 230 (100-351) | 0 (0-3) | 1059 (331-1667*) | 228 (53-340) | 213 (50-389) | 193 (77-455) |
| Day 14 | | 151 (21-363) | | 486 (195-550) | | N/A | | N/A | | N/A | | N/A | N/A | 376 (154-500) | 173 (49-267) | 147 (44-394) | 150 (65-281) |
| Day 28 | | 122 (7-247) | | 312 (170-433) | | 157 (98-311) | | 97 (44-203) | | 131 (43-173) | | 107 (52-179) | 0 (0-5) | 222 (91-364) | 55 (25-105) | 80 (28-175) | 74 (48-131) |
| Day 56 | | 104 (32-266) | | 162 (110-281) | | N/A | | N/A | | N/A | | N/A | N/A | 200 (53-331) | 28 (11-63) | 75 (27-178) | 60 (27-93) |
| Day 84 | | 126 (28-158) | | 104 (67-169) | | 79 (31-155) | | 53 (19-111) | | 84 (36-140) | | 67 (24-121) | 2 (0-5) | 155 (53-331) | 26 (9-56) | 46 (0-128) | 61 (23-105) |
| Day 168 | | 64 (11-108) | | 113 (51-174) | | 53 (25-109) | | 36 (18-101) | | 74 (20-116) | | 41 (12-81) | 1 (0-3) | 123 (8-252) | 19 (6-60) | 52 (8-68) | 44 (14-158) |
| Day 365 | | 78 (20-116) | | 70 (35-183) | | N/A | | N/A | | N/A | | N/A | N/A | 100 (36-127) | 10 (0-46) | 30 (17-72) | 49 (14-79) |
| >1000 days | | 54 (22-100) | | 170 (112-290) | | 43 (20-71) | | 53 (27-118) | | 79 (36-124) | | 96 (15-166) | 3 (0-7) | 84 (50-150) | 7 (1-26) | 31 (8-94) | 73 (14-112) |

Median Elispot responses (IQR), SFC per million PBMC. N/A, not applicable because timepoint was not included in trial.

*The upper limit of detection of the IFN-γ Elispot assay was 1667 SFC per million PBMC.
